# Learning to Swim: An Exploration of Negative Prior Aquatic Experiences Among Children

**DOI:** 10.3390/ijerph17103557

**Published:** 2020-05-19

**Authors:** Amy E. Peden, Richard C. Franklin

**Affiliations:** 1 Royal Life Saving Society, Sydney 2007, NSW, Australia; richard.franklin@jcu.edu.au; 2 College of Public Health, Medical and Veterinary Sciences, James Cook University, Townsville 4811, QLD, Australia; 3 School of Public Health and Community Medicine, UNSW Sydney, Kensington 2052, NSW, Australia

**Keywords:** drown, swim, physical education, child, swimming lessons, aquatic activity, teaching, prevention, safety, business

## Abstract

Learning to swim via a structured program is an important skill to develop aquatic competencies and prevent drowning. Fear of water can produce phobic behaviors counterproductive to the learning process. No research examines the influence of negative aquatic experiences on learning to swim. This study explored the influence of children’s negative prior aquatic experiences (NPAE) on learn-to-swim achievement via swim school data. Children’s enrolment records (5–12 years) in the Australian Capital Territory were analyzed via demographics, level achieved and NPAE. NPAE was recorded as yes/no, with free text thematically coded to 16 categories. Of 14,012 records analyzed (51% female; 64% aged 6–8 years), 535 (4%) reported a NPAE at enrolment. Males, children with a medical condition and attending public schools were significantly more likely (*p* = 0.001) to report a NPAE. Children reporting a NPAE achieved a lower average skill level at each year of age. The largest proportion (19%) of NPAE reported related to swimming lessons. NPAE have a detrimental influence on aquatic skill achievement. We recommend increased adult supervision to reduce likelihood of an NPAE occurring, while also encouraging swim instructors to consider NPAE when teaching swimming and develop procedures to ensure a NPAE does not occur during instruction.

## 1. Introduction

Drowning is one of the leading causes of accidental death among children [1]. For all ages, the current annual global estimate is 295,000 drowning deaths [2], although this figure is thought to underreport fatal drowning, in particular boating and disaster related drowning mortality [3]. Drowning disproportionately impacts children and young people, with over half of all drowning deaths occurring among people aged under 25 years [1]. In many countries, children under five years of age record the highest rate of fatal [4,5] and non-fatal drowning [6], with incidents commonly occurring in swimming pools [7] and bathtubs [8] in high income countries and in water bodies in and around the home in low income contexts [9]. 

Learning to swim has been found to be an effective drowning prevention strategy [10] and has been proposed by the World Health Organization (WHO) as one of ten key strategies for global drowning prevention [11]. Participation in formal swimming lessons has been shown to reduce drowning risk among children aged 1–19 years [12] and a recent review of evidence suggests that teaching aquatic competencies to young children causes no increased risk [13], particularly when combined with the additional drowning prevention strategies of supervision, restricting access to water and caregiver training in cardiopulmonary resuscitation (CPR) [14].

It has been posited that aquatic competencies are fundamental movements and skills gained in the formative years of a child’s lifespan are essential for safe aquatic participation throughout adulthood [15,16,17]. Swimming is the most popular out-of-school participation sport among Australian children aged 0–14 years [18]. However there has been recent discussion of the decline in swimming skills among Australian children [19], and reduced participation among those from low socio-economic backgrounds [20], potentially leading to increased risk of drowning in adulthood. 

**Figure 1:**
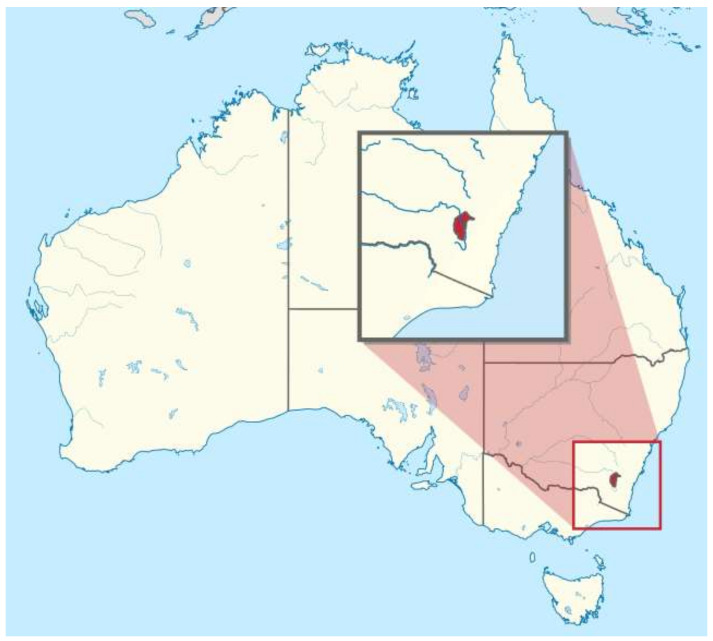
Map of Australia identifying the Australian Capital Territory (ACT). Note: Image used with permission under the Creative Commons Attribution-Share Alike 3.0 Unported license.

In Australia, learn-to-swim instruction is taught through multiple delivery modes including through schools, within vacation programs and through private instruction [21]. Australian is a federated country made up of six states and two territories. The Australian Capital Territory (ACT) is the smallest state or territory in Australia with a land area of 235,817 hectares [22]. The ACT is the second smallest population of any state or territory as at September 2019 of 428,060 [23] (Figure 1). The ACT population has little socio-economic disadvantage, with a median annual income 32% above the national average [22]. Therefore, barriers to participation in learn-to-swim previous identified, such as cost, pool space and distance to travel are not as much of an issue in the ACT as in other States and Territories in Australia [21].

Some recent learn-to-swim methods in young children have been criticized for instilling stress and fear which may be counterproductive to the learning process [24]. Such methods have prompted organizations such as the Australasian Council for the Teaching of Swimming and Water Safety (AUSTSWIM) to issue a policy opposing forced back float techniques in children, due to such techniques being harmful and not developmentally appropriate for infants and young children [24]. Fear of water has been shown to have negative lifestyle consequences [25] due to a persistence from childhood through to adulthood [26], as well as being transferred from parent to child [27]. 

Such negative prior aquatic experiences can result in water phobic behaviors [28]. The literature on such behaviors is minimal and, to date, has focused largely on fear of water in the context of showering and bathing [29,30] and fear of swimming and swimming pools [31]. The avoidance of swimming pools has received little attention, aside from a study exploring pool avoidance for an adolescent with autism [32]. However, a study by Chan et al., explored the impact of an intervention on three typically developing children aged 3–7 years who showed a fear of water [28]. While one of the three children had displayed a fear of water since birth, the other two children had fear from what we are terming negative prior aquatic experiences (NPAE). That is, two prior falls into swimming pools requiring rescue for one child and a fall into a swimming pool requiring rescue by a teacher [28] for the remaining child in the study. Chan et al., reported the phobic behaviors of the children involved in the study when participating in swimming lessons. Such behaviors included crying, verbal refusal to attempt skills, requesting a lesser skill when prompted to attempt a new skill, clinging to the shoulders of the swimming instructor and refusing to enter the pool if unable to stand [28]. Such behaviors are obviously counterproductive to the learning process and present a barrier to participation. 

Similarly, a study by Franklin et al., exploring factors affecting children’s achievement in a learn-to-swim program, has previously explored the influence of negative prior aquatic experiences on level achieved within the ACT learn-to-swim program [33]. This study found a negative prior aquatic experience achieved, on average, a lower level, being significantly lower for six- to nine-year-olds [33]. To build on this study, and to add to the sparse literature on this topic, the current study aims to explore the types of negative prior aquatic experiences reported by parents and caregivers of participants in the ACT learn-to-swim program and identify those more likely to be affected. The findings of this study are used to make recommendations for swimming teachers providing instruction during both private and school-based physical education (PE) lessons as well as program managers and administrators.

## 2. Materials and Methods

This study is a retrospective cross-sectional analysis of children’s participant records in a learn-to-swim program, including demographic and aquatic achievement data, with a particular focus on children’s negative prior aquatic experiences as reported by parents. In this study, the term learn-to-swim is a catch-all term covering a broad range of aquatic competencies that include rescue skills, swimming, floating, treading water and water safety knowledge among others. Methods used in this study are now discussed.

### 2.1. Data Source

Participant data used in this study was collected from an administrative dataset of Royal Life Saving Society—Australian Capital Territory (RLSS-ACT), which delivered the Swim and Survive Program in the ACT to primary school children (aged 5–12 years) during school hours. RLSS-ACT was responsible for the program including logistics, enrolment, transport, delivery, and reporting with financial support to subsidize the program provided by the ACT government.

The Swim and Survive program is a swimming and water safety program which sees participants progress through the ‘Wonder’ (6 to 36 months), ‘Courage’ (3 to 5 years) and ‘Active’ (5–14 years) components of the program which span 18 levels [34].

A parent or guardian of each child who participated in the program was required to provide a signed enrolment form. The enrolment form collected information on the child’s date of birth, sex, class (year level at school), negative prior aquatic experience, and current level of swimming ability. Data on negative prior aquatic experience were recorded on the enrolment form as a yes/no field followed by free text which ranged in detail from a couple of words (i.e., bad experience) to 1 line of text (i.e., *‘…When child 4 years old child’s father wasn’t supervising child at a hotel and child fell in to the deep end. 2 other adults jumped in and saved child…’ [Child’s Parent])*. Only data provided within the confines of this question on the enrolment form were able to be used for the negative prior aquatic experiences explored in this study. 

### 2.2. Data Cleaning and Coding

There were 15,234 participant records in the program database during the study period (1 January 2009, to 31 December 2012). There were 385 cases (2.5%) with unknown age and 102 participant records (0.7%) with ages outside 5–12 years that were removed from the dataset prior to analysis. A further 123 records (0.8%) where the participant’s sex was missing were also removed. As the intent of this paper was to explore negative prior aquatic experiences, the further 578 (4.0%) records which did state either yes or no to the question were further removed from the data. After this data cleaning, there remained 14,012 records for analysis.

Where questions were not completed, these were entered into the database as blank fields (i.e., missing). Where questions within the enrolment form were incomplete or unanswered, the variable within the record was deleted from the analysis. This approach is called complete-subject analysis and is a recognized data analysis technique to address missing information where the complete data are assumed to be a random sample of all of the participants in the study [35].

The data were entered into a FileMaker Pro database system used to help run the program. De-identified data (no names or address information) were extracted from the database into Microsoft Excel TM and coded for ease of analysis in SPSS V20 [36].

An enrolling participant’s pre-existing medical condition was also captured on the enrolment form. The presence of a medical condition was coded yes/no, and those medical conditions reported, were coded to ICD-10 Tabular List of Diseases [37]. School type was also classified as being public (i.e., wholly government funded) or private (e.g., independent or Catholic schools which students pay fees to attend). Due to ethical constraints, cell counts <4 were concealed using NP (not presented).

#### 2.2.1. Coding of Negative Prior Aquatic Experience(s)

The question that asked parents if the child has had a negative prior aquatic experience also asked parents to record details using free text of the negative experience, and these answers required coding. Negative prior aquatic experience was coded yes, no, unknown. Those who specified a negative prior aquatic experience were thematically coded into 16 categories using an inductive methodology as outlined by Braun and Clarke [38]. This five-phase methodology was undertaken as follows: Both authors separately familiarized themselves with the data by rereading the data and noting down initial themes (Phase 1). Both authors then separately generated initial codes by systematically coding interesting features of the entire dataset (Phase 2). Both authors then came together to cross-check and confirm individual codes and thematically determine categories (Phase 3). Separately, both authors sorted codes into categories (Phase 4). Both authors then came back together to compare the contents of each category and refine any outstanding issues (Phase 5).

These categories included “scared of deep water”, “negative experiences during swim lessons”, “fell in the water”, and “doesn’t like the water”. There was also an “other” category which included “panics when floating on the back”, “scared about jumping from heights”, and “doesn’t like being pushed past comfort zone” (Table 1). Sex has been removed from the example responses to de-identify them.

A coding framework was used, as follows: (1) preference given to the activity undertaken, (2) preference given to the aquatic location of the negative experience, and (3) other. For example—a child who experienced a non-fatal drowning due to a fall into a backyard pool was coded to “fell in”. A child who witnessed a man be resuscitated at the beach after a non-fatal drowning incident, was coded to “non-fatal drowning”. Typically however, non-fatal drowning cases often involved the child themselves. Where two negative prior aquatic experience were given i.e., “knocked over by waves at the beach and also got into difficulty at a leisure centre”, the first negative experience mentioned was the one used for coding, i.e., negative experience—beach.

For the top four most reported categories (negative experience swim lessons, fell in, non-fatal drowning and negative experience—beach), a sub-analysis of key themes was undertaken using the same five step approach to thematic analysis described above.

#### 2.2.2. Coding and Analysis of Achievement Level

For each record, the level of achievement in the final lesson was captured in the database. This was used with the child’s age to explore differences in average level achieved by age between those who did and didn’t report a negative prior aquatic experience.

### 2.3. Data Analysis

Data analysis was undertaken in SPSS V20 [36]. Descriptive and chi square analysis (*p* < 0.05) were used to explore the categories of negative prior aquatic experiences. For cell counts <5 a Fishers Exact test was conducted. Analysis of variance (ANOVA), with a 95% confidence interval, was used to calculate statistically significant (*p* < 0.05) differences for those children in the dataset with and without a negative prior aquatic experience and influence on level achieved. 

### 2.4. Ethics Approval

Ethics approval for this study was granted by the James Cook University Human Research Ethics Committee (HREC) (H7838) in 2019.

## 3. Results

From the 14,012 enrolment records available for analysis, a total of 535 participant records (3.8%) noted a negative prior aquatic experience. Of these, 56.4% were male and the mean age of the records reporting a negative experience was 8 years (M = 7.67 years (SD = 1.63)) (Table 2).

Males (X^2^ = 12.8; *p* = 0.001), children with a medical condition (X^2^ = 10.7; *p* = 0.001) and children attending public schools (X^2^ = 12.2; *p* < 0.001) were more likely to report a negative prior aquatic experience. Children aged 12 years were significantly less likely to report a negative prior aquatic experience (X^2^ = 4.8; *p* = 0.028) (Table 2).

The most common categories of negative prior aquatic experiences were as follows: negative experiences during swimming lessons (*n* = 99; 18.5%), fell in (*n* = 59; 11.0%), non-fatal drowning (*n* = 45; 8.4%) and negative experiences at beaches (*n* = 44; 8.2%) and in swimming pools (*n* = 36; 6.7%). Experiences recorded in the ‘other’ category included slips and trips on pool deck, being frightened by a bee while in the water and having a seizure while in the water (Table 1).

When exploring type of negative experience by sex, males were significantly more likely to have experienced a non-fatal drowning (X^2^= 5.7; *p* = 0.017), while females were more likely to have a negative prior aquatic experience associated with a family member (X^2^ = 0.5; *p* = 0.0457). There were no other differences identified in type of negative prior aquatic experience when explored by sex (Table 3).

Key subthemes were identified for the four most commonly reported negative prior aquatic experiences, namely, negative experience swim lessons, fell in, non-fatal drowning and negative experience—beach. For children who recorded a negative prior aquatic experience during a swimming lesson, the most commonly reported negative experiences were being submerged or dunked (*n* = 41; 41.4% of the 99 responses in the category), followed by instructor neglect leading to children needing to be rescued (*n* = 13; 13.1%) and children being pressured to attempt skills against their will (*n* = 13; 13.1%). Experiences coded to the ‘other’ category included issues around not enough instructors, children swimming away from an instructor during a lesson and children jumping in before instructors were ready (Table 4).

Key subthemes identified within the non-fatal drowning category were as follows: deep water (17.8%; *n* = 8); non-fatal drowning in a pool (8.9%; *n* = 4) and the child reported as being unable to resurface in 6.7% of cases (*n* = 3). No further details were given in 57.8% of non-fatal drowning cases (*n* = 26). Of note, 15.6% (*n* = 7) of the responses in the non-fatal drowning category were reported as occurring when the child was between 1 and 4 years of age (data not shown).

Of the 59 negative prior aquatic experiences which occurred as a result of falls into water, the leading activity was a fall into a swimming pool (*n* = 35; 59.3%). Additional key subthemes within the negative experience—beach category were children getting caught in rips (*n* = 16; 36.4%) or dumped by waves (*n* = 16; 36.4%) (data not shown).

**Figure 2:**
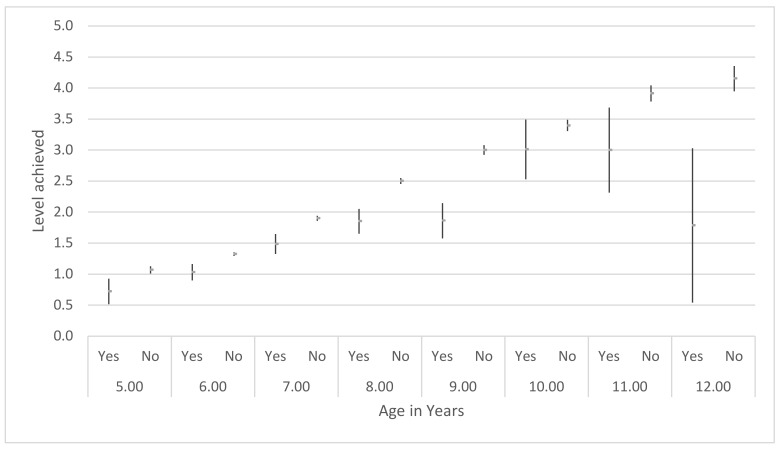
Average level achieved by age in years and negative prior aquatic experience yes/no.

Children who reported a negative prior aquatic experience recorded a lower average level achieved at every year of age, when compared to children of the same age who did not record a negative prior aquatic experience. This difference was statistically significant for all ages except for those aged ten years. The difference was most pronounced at 12 years of age, with children with a negative prior aquatic experience achieving an average level of 1.8 compared to an average level of 4.2 for those without such an experience (Figure 2).

## 4. Discussion

This study, the first of its kind, explored negative prior aquatic experiences reported by parents and caregivers of children participating in a primary school learn-to-swim program. This study found 4% (*n* = 535) of enrolment records reported a negative prior aquatic experience. Males, children with a medical condition and children attending public schools were significantly more likely (*p* = 0.001) to report a negative prior aquatic experience. The largest proportion (19%) of negative prior aquatic experiences reported related to swimming lessons. Children with a negative prior aquatic experience achieved a lower level in the program, on average, than those who did not report one.

While learning to swim is a life-skill and may reduce drowning risk [11,12], a negative prior aquatic experience creates fear which in turn can lead to water phobic behaviors [28], negatively influencing the achievement of children while participating in learn-to-swim programs [33]. Thus, it is important to explore the types of negative prior aquatic experiences being reported for children in order to reduce such experiences from occurring. This study has identified several key themes including implications for learn-to-swim providers and opportunities for future research, which are now discussed. 

### 4.1. Negative Prior Aquatic Experiences and Those More Likely to be Affected

A key finding of this study was identifying factors which impacted likelihood of a negative prior aquatic experience being reported. Analysis indicated those attending public schools (X^2^ = 12.2; *p* < 0.001) and having a pre-existing medical condition (X^2^ = 10.7; *p* = 0.001) were factors which significantly increased the likelihood of reporting a negative prior aquatic experience. It is vital that all parents and caregivers put strategies in place, such as active adult supervision [39], to reduce the likelihood of a negative prior aquatic experience occurring. However, our findings suggest there may be a need to specifically focus on these at-risk groups. Further research is also required to better understand the reasons behind why these groups are at increased risk of a negative prior aquatic experience. 

The authors postulate that those attending public schools may represent children of a lower socio-economic status than those likely to attend private schools. Social determinants of health [40], such as socio-economic status are a key indicator of injury risk [41,42], including drowning [43], indicating a need for focused prevention efforts to target communities at increased risk. 

Similarly, for those with pre-existing medical conditions, previous research has indicated an increased risk of drowning for children with epilepsy [44] and autism spectrum disorder (ASD) [45,46]. Children with medical conditions may therefore be doubly at risk of drowning, with increased risk due to their condition, and negative prior aquatic experiences impacting their willingness to participate in swimming lessons and their ability to achieve required competencies. It is vital that children with pre-existing medical conditions learn-to-swim in lessons with appropriate safety measures in place, such as appropriate teacher to student ratios and are taught by instructors trained in teaching swimming to children with such conditions [47]. Additionally, parents of children with medical conditions that increase drowning risk must be counselled on this and strategies to reduce the risk, such as learning to swim and adult supervision at all times in and around water. Teachers are a powerful portal for the provision of information to parents of children attending lessons [48,49], however other strategies will be needed to reach parents of children not enrolled in lessons, or who have ceased lessons before minimum skills have been achieved. Further research is required to determine if some negative prior aquatic experiences are due to children with pre-existing medical conditions being unable to keep up with peers and/or progress through lessons as their peers do.

### 4.2. The Impact of a Child’s Sex on Type Negative Prior Aqutic Experiences

Study findings indicate that males were significantly more likely (X^2^ = 12.8; *p* = 0.001) to report a negative prior aquatic experience when compared to females. Similarly, this study has identified differences in the type of negative prior aquatic experience reported by children based on sex. Males were more likely to report a negative prior aquatic experience as a result of a non-fatal drowning (X^2^ = 5.7; *p* = 0.017), while females were more likely to have a negative experience relating to a family member (X^2^ = 0.6; *p* = 0.0457). Research indicates males are more likely to experience fatal and non-fatal drowning requiring hospitalization [4,6,50] and while reduced supervision may be a factor, sex has not been found to impact supervision provided to young children (ages 2–5 years) [51]. Further research is required into supervisory practices of older children and to explore the link between negative prior aquatic experiences of family members and influence upon female children.

### 4.3. Negative Experiences during Swimming Lessons

Of concern for learn-to-swim teachers and swim schools, were the 19% of negative prior aquatic experiences recorded as occurring during learn-to-swim lessons. Such experiences ranged from being submerged or dunked in water before the child was ready, instructor neglect leading to children needing to be rescued and children being pushed to attempt skills against their will. 

Such experiences should not occur during swimming lessons and teacher training must emphasize the need for empathy for students [52] in tandem with appropriate supervision and teaching techniques. The findings of this study should be used to ensure the prioritization of child-centered approaches to learning to swim [53], thereby reducing the likelihood of children experiencing negative prior aquatic experiences, such as those identified in this study.

### 4.4. Other Negative Prior Aquatic Experiences

In addition to negative prior aquatic experiences incurred during swimming lessons, other common types of negative prior aquatic experiences included falls into water, non-fatal drowning and negative experiences at beaches and swimming pools. It is not as clear how these children fell into water however, these findings suggest parents and caregivers should be mindful of a child’s skill level when taking them to aquatic locations. It is important that parents and caregivers do not overestimate children’s skills because they are enrolled in swimming lessons [49]. Similarly, it is important that parents and caregivers are educated on the unique hazards and risks that different aquatic locations pose [48].

What is clear from the bad experiences incurred, in particular non-fatal drowning and children getting into trouble in deep water at rivers, falling into pools and being caught in rips at beaches, is the need for active adult supervision of children when in and around water [39]. While supervision is a well-understood drowning prevention strategy for children under five [54]; supervision strategies must continue, and be adapted, for older children. Like supervision policies adopted at aquatic facilities, such as the Keep Watch @ Public Pools program [55,56]; supervision can shift from being in the water and within arm’s reach for children under five and non-swimmers to supervising from outside the water while dressed ready to enter the water if needed, to regularly checking up on children in the pool for pre-teens and adolescents. Similarly, it is important that parents and caregivers are aware that supervisory practices should be based on both age of child and a child’s ability [55].

### 4.5. Influence of Negative Prior Aquatic Experiences on a Child’s Learn-to-Swim Journey

This study identified negative prior aquatic experiences adversely impact a child’s ability to learn aquatic competencies, resulting in a lower average level achieved at every year of age when compared to children of the same age who did not report a negative prior aquatic experience. Those involved in learn-to-swim instruction must be mindful of the diversity of negative prior aquatic experiences, including a fear of drowning [57], which may present as water phobic behaviors [28] significantly impacting a child’s ability to learn-to-swim. Swim instructors should work with parents of children who report a negative prior aquatic experience, to collaboratively develop strategies to address the child’s particular concerns, recognizing that an increased level of support (i.e., smaller class sizes, slower pace of instruction or varied lesson delivery) may be required early on in the child’s learn-to-swim journey. It is important that the issue of negative prior aquatic experiences is communicated to parents and they are provided with the opportunity to share information on their child’s negative prior aquatic experiences without shame. This data, should be kept confidential, and shared only with program managers and the impacted child as needed.

Further exploration on the influence of a negative prior aquatic experience on enrolment and maintenance of participation in learn-to-swim instruction is required. Data analyzed in this study appears to indicate that older children (i.e., those aged 12 years) were significantly less likely (X^2^ = 4.8; *p* = 0.028) to report a bad experience. This does not take into account that children with negative prior aquatic experiences may be exiting lessons early. These children may be exposing themselves to increased risk of drowning throughout adulthood due to a lack of swimming and water safety skills. Further population level research is required to explore this further. Learn-to-swim providers and swim school managers should be encouraged to include a section on their enrolment form to capture information on an enrolling child’s negative prior aquatic experience(s) and to transmit this information to the child’s future instructors. Consideration should be given to opportunities for parents and caregivers to update this information periodically, as a negative experience occurs and/or the influence of a negative prior aquatic experience declines over time. 

### 4.6. Future Research Opportunities

There are a range of challenges associated with teaching children who have had a negative prior aquatic experience, which are likely to impact enrolment, learning and maintenance of participation in learn-to-swim. Similarly, research has shown that parents may project their own fears or negative experiences with water onto their children [27]. What is considered a negative prior aquatic experience will likely differ between and among parents. It may also be the case that some parents may not disclose a negative prior aquatic experience if their child is resilient and appears to be unaffected as a result of the experience. Further research is required to explore what parents consider a negative prior aquatic experience. Cause and effect around child’s ability and negative prior aquatic experiences also needs to be explored in further detail, i.e., a child’s negative prior aquatic experience is known to adversely impact a child’s ability to achieve aquatic competencies [33] but if a child is struggling to learn-to-swim, does this also lead to a negative prior aquatic experience? Prospective studies which gather data on negative prior aquatic experiences from both parent/caregiver and child are required. Similarly, longitudinal studies may also add value to identify ongoing influences of a negative prior aquatic experience on children as they grow. Research should also explore if, and how, children recover from negative prior aquatic experiences and which forms of additional support or teaching techniques during learn-to-swim instruction are most effective at overcoming these experiences. Larger studies would allow for the statistical power to explore impact of specific categories of negative prior aquatic experiences on skill achievement. 

### 4.7. Strengths and Limitations

This study reports unique data from a large dataset (>14,000 records) in a defined population of young children learning to swim. There was previously no understanding of NPAE in the published literature and as such data for this study was collected using a free text field on an enrolment form. This study advances our understanding and has identified categories of NPAE which may assist in streamlining future data collection.

However, the study is not without limitations. This study represents the reported data of parents and caregivers of children enrolled, as such, this relies upon the knowledge, willingness and accuracy of parents in reporting such negative prior aquatic experiences at time of enrolment. The data records the age at which the negative prior aquatic experience was reported and not when it was experienced. As such, responses may be subject to recall bias [58].The data may be subject to data entry errors and there may be duplicate records, representing the same child who participated in the program in multiple years. It is not known, however, if this resulted in duplicate negative prior aquatic experiences being recorded (i.e., parents reporting the same negative experience each year the child is enrolled or upon first enrolment). As data were de-identified, it was not possible to control for potential duplicate records. The coding framework employed influenced how negative prior aquatic experiences were coded. An alternative process may potentially result in different classifications. Although a large dataset, this study represents only one territory in Australia with a reasonably small and affluent population and studies drawing data from a national level, or comparative studies in other countries, are recommended. The transferability of findings to contexts dissimilar to Australia may be limited. However, while experiences of learning to swim may differ around the globe, the issue of negative aquatic experiences is highly likely to be universal. The data does not include information on children who dropped out of the program, future follow-up of these children is warranted. As data were de-identified it was not possible to contact parents for more information. As the provision of data were limited to the confines of the enrolment form, further prospective and qualitative research is proposed to gain a detailed understanding of the context within which these bad experiences occurred. Longitudinal studies are also recommended to identify ongoing influence of these negative prior aquatic experiences on children as they grow.

## 5. Conclusions

Negative prior aquatic experiences can lead to fear which presents as water phobic behavior which influences ability to learn-to-swim. Findings indicate negative prior aquatic experiences persist across age groups; boys, children attending public schools and those with a pre-existing medical condition were more likely to report a negative prior aquatic experience; and that negative prior aquatic experiences adversely influence a child’s ability to learn aquatic competencies, resulting in a lower average level achieved when compared to children of the same age who did not report a negative prior aquatic experience. To reduce the likelihood of a negative aquatic experience occurring, parents/caregivers and swim instructors should use strategies such as improved supervision to reduce the potential impact of such experiences on willingness and ability to learn-to-swim. Learn-to-swim providers should encourage parents and caregivers to report information on negative prior aquatic experiences at point of enrolment and provide an opportunity for parents and caregivers of those enrolled in lessons to update this information as and when a negative aquatic experience occurs subsequent to enrolment. Improving swim teachers’ skills, including empathy, to manage and prevent negative aquatic experiences during swimming instruction is also necessary. Further prospective research is required to explore the issue of negative prior aquatic experiences from both a child and parent/caregiver point of view. Longitudinal studies would also be valuable to identify any ongoing influences of such negative experiences on children as they grow. Learning to swim is a recognized drowning prevention strategy and reducing any barriers to the learning of such skills is vital to maximize a child’s ability to enjoy aquatic locations safely throughout their lifespans.

## Figures and Tables

**Table 1:** Categories of negative prior aquatic experiences and examples.

Negative Prior Aquatic Experience Categories	Negative Prior Aquatic Experience Examples
Negative experience swim lessons	“Nearly drowned at non-school lesson when instructor neglected to ensure safety” “Overwhelmed in last swimming lesson, group was too big-not enough instructors” “First swim teacher nearly drowned child by being inattentive while holding child under water. Is quite tentative about swimming”
Fell in	“At age 4 fell into a pool and had to be pulled out. It had frightened them” “Fell into a pond-very shaken” “Fell into shallow water when young. Still experiences levels of anxiety at public pools”
Non-fatal drowning	“Nearly drowned twice. Once during camp, once during lessons in 2010” “Nearly drowned at grandparents place 4 years ago. No CPR needed” “Near drowning at a swimming lesson age 3”
Negative experience—beach	“Carried away by a rip” “Dumped by a large wave several years ago”
Negative experience—swimming pool	“Walked down steps into a pool and slipped, couldn’t swim at the time “Was playing in the pool and couldn’t move forward so panicked”
Fear of water/doesn’t like the water	“Apprehensive about entering water”
Scared deep water	“Jumped in deep end of pool and couldn’t get to the surface, was rescued by older brother-affected water confidence” “Jumped in deep water, almost drowned” “Slipped under water when 3, lacks confidence when not able to touch bottom”
Other	“After lessons slipped and hit head on the floor. Fears might drown” “Got a fright from a bee on the water, slipped back and went under water while gasping in fright. Took a lung full of water, went into shock and sank to the bottom. Now has trouble with water over head” “Had a seizure in the water”
Scared when head under water	“Has a fear of getting face wet”
Negative experience flotation aid	“Fell off a flotation device about 2.5 years ago—had to be rescued”
Negative experience family member	“Father had a heart attack and died while they were swimming together” “Was thrown in by an adult who thought child could swim about 3 years ago. Is now careful in water and a little scared” “Older sister drowned–child was present at the time”
Scared lack of skill	“Still hesitant as very limited skills” “Is anxious of what will be asked of her, hates swimming lessons, lacks confidence”
Negative experience river/creek	“When very young had a scare in the river” “Got out of depth in a creek and was underwater. Made child nervous about not being able to touch the bottom”
Negative experience child	“Another child swam into child and pushed child into the wall causing child to chip tooth”
Negative experience water slide	“After going down a waterslide the person behind child landed on top of them and held child under” “Capsized at the end of a water slide on a rubber tube with dad”
Unknown	No further details given

Abbreviations: CPR = Cardiopulmonary Resuscitation.

**Table 2:** Demographics of participants by negative prior aquatic experience yes/no, chi square (*p* value), Australian Capital Territory (ACT), 20092012-(N = 14,012).

	Total		Negative Prior Aquatic Experience-Yes		Negative Prior Aquatic Experience-No		X^2^ Comparing Negative Experience Yes to Negative Experience-No (*p* Value)
	N	%	N	%	N	%	
Total							
	14,012	100.0	535	3.8	13,477	96.2	-
Sex							
Male	6848	48.9	302	4.4	6546	95.6	12.777 (0 = 0.001)
Female	7164	51.1	233	3.3	6931	96.7	
Age in Years							
5 years	625	4.5	27	4.3	598	95.7	0.449 (*p* = 0.503)
6 years	2938	21.0	127	4.3	2811	95.7	2.577 (*p* = 0.108)
7 years	3053	21.8	118	3.9	2935	96.1	0.023 (*p* = 0.878)
8 years	2905	20.7	106	3.6	2799	96.4	0.286 (*p* = 0.593)
9 years	1869	13.3	74	4.0	1795	96.0	0.117 (*p* = 0.732)
10 years	1386	9.9	51	3.7	1335	96.3	0.080 (*p* = 0.777)
11 years	835	6.0	25	3.0	810	97.0	1.642 (*p* = 0.200)
12 years	401	2.9	7	1.7	394	98.3	4.828 (*p* = 0.028)
Pre-existing medical condition							
Yes	1578	11.3	99	6.3	1479	93.7	10.674 (*p* = 0.001)
No	1556	11.1	58	3.7	1498	96.3	
Unknown	10,878	77.6	378	3.5	10,500	96.5	-
Type of school							
Public	10,236	73.1	426	4.2	9810	95.8	12.213 (*p* < 0.001)
Private	3776	26.9	109	2.9	3667	97.1	

NP = Not Presented.

**Table 3:** Category of negative prior aquatic experience by sex.

Negative Prior Aquatic Experience Category	Total	Sex				X^2^ (*p* Value)
		Male		Female		
	*n*	*n*	%	*n*	%	
Negative experience swim lessons	99	52	52.5	47	47.5	0.761 (*p* = 0.383)
Fell in	59	31	52.5	28	47.5	0.412 (*p* = 0.521)
Non-fatal drowning	45	33	73.3	12	26.7	5.698 (*p* = 0.017)
Negative experience—beach	44	25	56.8	19	43.2	0.003 (*p* = 0.959)
Negative experience—swimming pool	36	19	52.8	17	47.2	0.212 (*p* = 0.646)
Fear of water/Doesn’t like the water	31	19	61.3	12	38.7	0.314 (*p* = 0.575)
Scared deep water	30	13	43.3	17	56.7	2.224 (*p* = 0.136)
Other	27	18	66.7	9	33.3	1.208 (*p* = 0.272)
Scared when head under water	24	12	50.0	12	50.0	0.425 (*p* = 0.514)
Negative experience flotation aid	14	10	71.4	4	28.6	0.288 (*p* = 0.193) *
Negative experience family member	11	5	45.5	6	54.5	0.552 (*p* = 0.0457)
Scared lack of skill	11	7	63.6	4	36.4	0.763 (*p* = 0.435) *
Negative experience river/creek	9	6	66.7	NP	33.3	0.738 (*p* = 0.394) *
Negative experience child	7	4	57.1	NP	42.9	1.000 (*p* = 0.639) *
Negative experience water slide	7	4	57.1	NP	42.9	1.000 (*p* = 0.639) *
Unknown	81	44	54.3	37	45.7	0.176 (*p* = 0.675)

NP—not presented, *—Fisher’s exact test.

**Table 4:** Thematic breakdown of negative prior aquatic experiences during swimming lessons (*n* = −99).

**Category of Negative Prior Aquatic Experiences during Swimming Lessons**	***n***	**%**
Submerged or dunked	41	41.4
Instructor neglect	13	13.1
Pressured to attempt skills against child’s will	13	13.1
Unhappiness/stress	10	10.1
Not specified	9	9.1
Other	9	9.1
Change in teacher	4	4.0
